# Abundance, diversity, and feeding behavior of coral reef butterflyfishes at Lord Howe Island

**DOI:** 10.1002/ece3.1208

**Published:** 2014-09-02

**Authors:** Morgan S Pratchett, Andrew S Hoey, Christopher Cvitanovic, Jean-Paul A Hobbs, Christopher J Fulton

**Affiliations:** 1 ARC Centre of Excellence for Coral Reef Studies, James Cook UniversityTownsville, Queensland, 4811, Australia; 2 Oceans and Atmosphere Flagship, CSIROHobart, Tasmania, 7000, Australia; 3 ARC Centre of Excellence for Coral Reef Studies, Research School of Biology, Australian National UniversityCanberra, Australian Capital Territory, 0200, Australia; 4 Department of Environment and Agriculture, Curtin UniversityPerth, Western Australia, 6845, Australia

**Keywords:** Chaetodontidae, corallivore, disturbance, ecological function, endemism, schooling, selectivity, specialization

## Abstract

Endemic species are assumed to have a high risk of extinction because their restricted geographic range is often associated with low abundance and high ecological specialization. This study examines the abundance of *Chaetodon* butterflyfishes at Lord Howe Island in the south-west Pacific, and compares interspecific differences in local abundance to the feeding behavior and geographic range of these species. Contrary to expected correlations between abundance and geographic range, the single most abundant species of butterflyfish was *Chaetodon tricinctus*, which is endemic to Lord Howe Island and adjacent reefs; densities of *C. tricinctus* (14.1 ± 2.1 SE fish per 200m^2^) were >3 times higher than the next most abundant butterflyfish (*Chaetodon melannotus*), and even more abundant than many other geographically widespread species. Dietary breadth for the five dominant butterflyfishes at Lord Howe Island was weakly and generally negative correlated with abundance. The endemic *C. tricinctus* was a distinct outlier in this relationship, though our extensive feeding observations suggest some issues with the measurements of dietary breadth for this species. Field observations revealed that all bites taken on benthic substrates by *C. tricinctus* were from scleractinian corals, but adults rarely, if ever, took bites from the benthos, suggesting that they may be feeding nocturnally and/or using mid-water prey, such as plankton. Alternatively, the energetic demands of *C. tricinctus* may be fundamentally different to other coral-feeding butterflyfishes. Neither dietary specialization nor geographic range accounts for interspecific variation in abundance of coral reef butterflyfishes at Lord Howe Island, while much more work on the foraging behavior and population dynamics of *C. tricinctus* will be required to understand its’ abundance at this location.

## Introduction

Endemic species are an important component of biodiversity but are also considered to be disproportionately affected by disturbances, and more likely to go extinct because relatively moderate disturbances can simultaneously affect the entire population (McKinney 1997; Gaston 1998; Roberts et al. 2002). Moreover, geographic range is often correlated with abundance (e.g., Lawton 1993; Gaston 1994, 1996; Brown et al. 1995; McKinney 1997), further increasing the risk of extinction for restricted range species (Gaston et al. 1997; Gaston 1998). This double jeopardy of extinction risk may also be further compounded if small range size is associated with other traits (e.g., ecologically specialization and low dispersal: Gaston et al. 1997; Malcolm et al. 2006; Pimm et al. 2014), making these species even more vulnerable to extinction (Davies et al. 2004; Brook et al. 2008; Olden et al. 2008).

Ecological specialization (the extent to which species specialize in their use of prey or habitat resources) is increasingly considered alongside population size and geographical range as a key determinant of extinction risk (e.g., McKinney 1997; Davies et al. 2004; Dulvy et al. 2004; Koh et al. 2004; Brook et al. 2008). Ecological theory (e.g., Brown 1984) suggests that specialized species should have narrower geographic ranges and be less abundant than generalist counterparts, but empirical data (e.g., Gaston et al. 1997; Manne and Pimm 2001; Päivinen et al. 2005; Reif et al. 2006; Hobbs et al. 2010, 2011; Berkström et al. 2012) does not always support the theory. An alternative explanation is that extinction filtering promotes persistence of species with compensatory relationships between range size, ecological specialization and population size that reduce the risk of extinction (e.g., Johnson 1998; Williams et al. 2006).

Despite the importance of ecological specialization for the biology, ecology and evolution of animals (e.g., Futuyma and Moreno 1988), ecological specialization is either rarely or poorly quantified (Devictor et al. 2010). Coral-feeding butterflyfishes (*Chaetodon*; Chaetodontidae) are an ideal group to study ecological specialization because their feeding behavior and dietary composition is easily measured, as is the differential availability of alternative prey (e.g., Berumen et al. 2005; Blowes et al. 2013; Noble et al. 2014). This enables direct estimates of dietary specialization across gradients of prey availability (e.g., Lawton et al. 2012), clearly distinguishing species that display distinct preferences regardless of prey availability (fundamental or obligate specialists) versus those that vary in their patterns of prey use simply to make use of locally abundant prey types (realized or facultative specialists). Moreover, sympatric butterflyfishes often exhibit significant variation in dietary selectivity, ranging from species that feed almost exclusively on just one coral species (e.g., *Chaetodon trifascialis*, Pratchett 2005; Pratchett et al. 2013a) to species that feed on >50 coral species, often in direct accordance with their relative abundance (e.g., *Chaetodon lunulatus*, Pratchett 2005).

Butterflyfishes are among the best-studied group of coral reef fishes (Pratchett 2014), owing partly to their inherent reliance on live coral for food and associated vulnerability to significant and widespread declines in live coral cover (e.g., Wilson et al. 2006, 2014). Pratchett et al. (2008, 2011) showed that interspecific differences in the vulnerability of butterflyfishes to coral loss are greatest among species for which corals represent >80% of total food intake (termed obligate corallivores, Cole et al. 2008). However, even among obligate coral-feeding fishes, responses to coral loss vary depending upon the extent to which species are more or less specialized in their use of different coral prey (Pratchett et al. 2008). There is, therefore, a definite need to better understand the specific foraging behavior and ecological specialization of coral reef butterflyfishes, especially among those species that are geographically restricted and exposed to local coral depletion (Lawton et al. 2012).

In this study, we explore the abundance, diversity and feeding behavior of *Chaetodon* butterflyfishes Lord Howe Island, and assess whether local abundance of individual species is related to their dietary specialization and/or geographic range. Lord Howe Island is the world’s southernmost coral reef, with fish faunas comprising a mix of both tropical and temperate species (Zann 2000), and a relatively high number of endemics (Randall 1976). Previous studies conducted within tropical coral-dominated environments have revealed that specialist coral-feeding species tend to dominate butterflyfish assemblages (Emslie et al. 2010; Pratchett et al. 2013a), but coral-feeding fishes are under-represented at some marginal or peripheral coral reef locations (e.g., Pratchett et al. 2013b). Given high cover of corals across much of the reef habitat at Lord Howe Island (Hoey et al. 2011), we would expect to find a high abundance of coral-feeding butterflyfishes, though the isolation and extreme latitude may moderate the abundance of some species. In this study, direct feeding observations were used to quantify both feeding rates and diet (or feeding substrata) of dominant butterflyfishes. Notably, this is the first study on the feeding habits of the three-striped butterflyfish (*Chaetodon tricinctus*), which is endemic to Lord Howe Island and nearby reefs (Hobbs et al. 2009; van der Meer et al. 2013).

## Methods

### Field surveys

**Figure 1:**
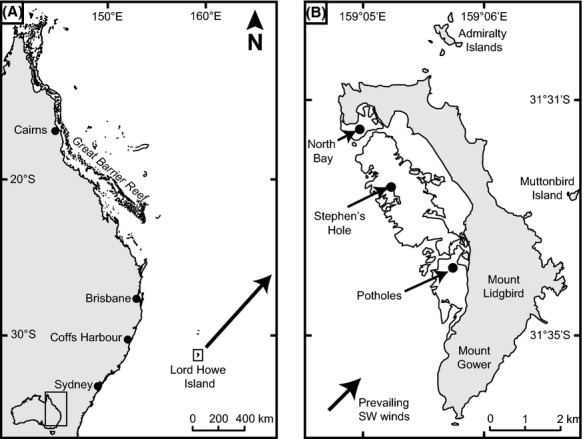
Map showing (A) geographic location of Lord Howe Island, and (B) the location of the three study sites on Lord Howe Island (North Bay, Stephen’s Hole, and Potholes) used to quantify butterflyfish assemblages.

Lord Howe Island (31°32′S, 159°04′E) is located 630 km east of the Australian mainland in the Tasman Sea (Fig. 1A). The western side of the island is dominated by an extensive lagoon with a high cover (*ca*. 30%), but low diversity, of scleractinian corals (e.g., Hoey et al. 2011). Sampling for this study was undertaken at three sites (North Bay, Stephen’s Hole and Potholes) equally spaced along the lagoon in areas of distinct platform reef <2 m depth, separated by deeper (4–6 m) sandy areas (Fig. 1B).

Butterflyfish abundance was quantified using underwater visual census (UVC) along haphazardly placed 50 × 4 m belt transects (*n* = 12 replicates per site) in December 2011. Butterflyfishes were surveyed while simultaneously deploying a 50-m transect tape to delineate transect length. All butterflyfishes 2 m either side of the transect midline were then recorded to species, as well as estimating their total length (TL, to nearest cm) and recording group size. Coral cover and benthic composition were quantified using point-intercept transects (following Pratchett et al. 2004, 2011) to record the specific substratum type underlying uniformly spaced points (0.5 m apart) along the length of each 50 m transect. Scleractinian (hard) corals, alcyonacean (soft) corals, and macroalgae (>5 mm) were identified to genus (and *Acropora* hard corals were further defined to tabulate or arborescent growth forms), with other substratum types categorized as sand/rubble or pavement.

### Feeding observations

To characterize and compare the feeding rates and diets of butterflyfishes at Lord Howe Island, the range of prey types, and the proportional use of different prey types by each species of butterflyfish (use was defined as an observed bite by the individual on a prey type), was quantified using replicate 3-min feeding observations following Pratchett (2005). Feeding observations were conducted during a similar time of year in each two consecutive years, May 2010 and June 2011. Feeding observations only commenced after the focal individual had taken their first bite, or 3-min after the observation started to allow fish to acclimate to observer presence. Observations were aborted if the focal individual fled or sought shelter from the observer. During each feeding observation, the total number of bites taken from different genera of hard coral, soft coral or any other noncoral macroinvertebrate was recorded. For the dominant coral genera, *Acropora*, we also distinguished between tabular (e.g., *Acropora glauca*), and arborescent (e.g., *Acropora yongei*) colonies. The number of bites taken from other reef substrata (i.e., consolidated reef pavement, coral rubble, or sand) that were not obviously occupied by corals or macroinvertebrates was also recorded. A minimum of 20 feeding observations were conducted for each of the five most common butterflyfish species recorded at Lord Howe Island: *C. lunulatus*, *Chaetodon melannotus, Chaetodon plebeius*, *C. tricinctus,* and *C. trifascialis*. Increased sampling effort was applied to the endemic *C. tricinctus* (186 of 419 feeding observation) due to apparent size-based differences in feeding behavior (discussed below).

### Data analyses

Spatial variation in the abundance and composition of *Chaetodon* butterflyfishes and categories of reef substratum were examined across the three sample sites (North Bay, Stephen’s Hole, and Potholes) using permutational multivariate analysis of variance (PERMANOVA). PERMANOVAs were conducted with 9999 permutations of the raw data constructed into resemblance matrices for the *Chaetodon* assemblages using a modified Gower Log_10_ measure (Anderson et al. 2006), and for the reef substratum categories using a Bray-Curtis similarity measure on square-root transformed data for the 36 transects (Anderson et al. 2008). Ordinations were used to visualize structure within the reef substratum and *Chaetodon* assemblages via principal coordinates analysis (PCO) on the same resemblance matrices. Pairwise PERMANOVA was used to further explore differences between sites. PCOs were optimized with vector overlays of raw Pearson correlations (limited to *r* > 0.4) and bubble plots to explore key *Chaetodon* species and substratum categories underlying spatial structure in this reef assemblage.

The extent to which spatial differences in *Chaetodon* assemblages could be explained by reef habitat composition was explored by distance-based linear models (DISTLM), which were based on the same resemblance matrices above, and used Akaike Information Criteria for finite samples (AICc) to select the “best” models with a range of settings (models with either 1, 2, 3, or 4 substratum categories incorporated) from all of the possible combinations of habitat predictor variables (Anderson et al. 2008). As recommended by Anderson et al. (2008), we checked for multicollinearity among possible habitat predictor variables using draftsman plots. This led to exclusion of abiotic substratum categories (sand/rubble, pavement) from the DISTLM analysis, as they were strongly (negatively) correlated with biotic categories (chiefly scleractinian corals). All analyses and ordinations were performed in PRIMER (version 6.1.16) with PERMANOVA+ (version 1.0.6).

To compare dietary composition and feeding selectivity among *Chaetodon* butterflyfishes, forage ratios were calculated following Manly et al. (2002), which illustrate the use of each prey category (number of bites taken) relative to the availability of each prey type across the three study sites. Bonferroni-corrected 95% confidence limits were calculated for each prey category used by each butterfly- fish species to establish the significance of prey selectivity. Selection ratios −95% CI that were >1 indicate that prey that were used significantly more than expected based on their availability (i.e., preferred), while ratios +95% CI that were <1 indicate prey that were used disproportionately less than expected (i.e., avoided).

Variation in both bite rates and diet breadth were analyzed using two-way ANOVAs to detect differences among species (*C. lunulatus*, *C. melannotus, C. plebeius*, *C. tricinctus,* and *C. trifascialis*) and among locations (North Bay, Stephen’s Hole, and Potholes), and Tukey’s post hoc test was used to reveal major differences among species. Raw data on the number of bites taken by each individual butterflyfish were square-root transformed prior to analyses to reduce the influence of occasional very large values. Replicate estimates of diet breadth were based on the number of distinct coral types that were consumed by each individual during the 3-min feeding observation; specialist species are expected to concentrate feeding on only 1–2 coral species, whereas generalists may feed on predominant or preferred prey while actively foraging across a range of different prey types (Pratchett 2014). One-way ANOVA was used to test for size-related differences in feeding rates for *C. tricinctus*, comparing among individuals with an estimated TL of <5 cm, 5–10 cm, and >10 cm. It was apparent during feeding observations that bite rates were highest among the smallest size classes and tended to decline with increasing size, so a minimum of 20 feeding observations were conducted for each size class. Similar analyses were not performed for other *Chaetodon* butterflyfishes, mainly because there was much less variation in the size of fishes, and so most feeding observations were of larger (presumably adult) individuals.

After accounting for spatial variation in abundance of different butterflyfishes, overall abundance of each species was determined by averaging across all sites. This aggregate measure of individual abundance was then used to examine whether interspecific differences in local abundance are related to geographic range (across all species present) and diet breadth (for subset of species for which dietary composition was measured). To compare geographic range among butterflyfishes, we used published estimates of maximal area of occurrence (Jones et al. 2002). Diet breadth was calculated as described above.

## Results

**Figure 2:**
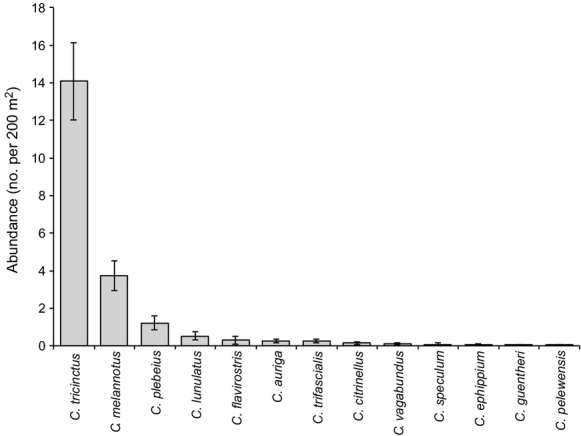
Mean (±SE) abundance of all *Chaetodon* butterflyfishes recorded at Lord Howe Island. Data are pooled across all sites to highlight relative abundance of different species.

**Figure 3:**
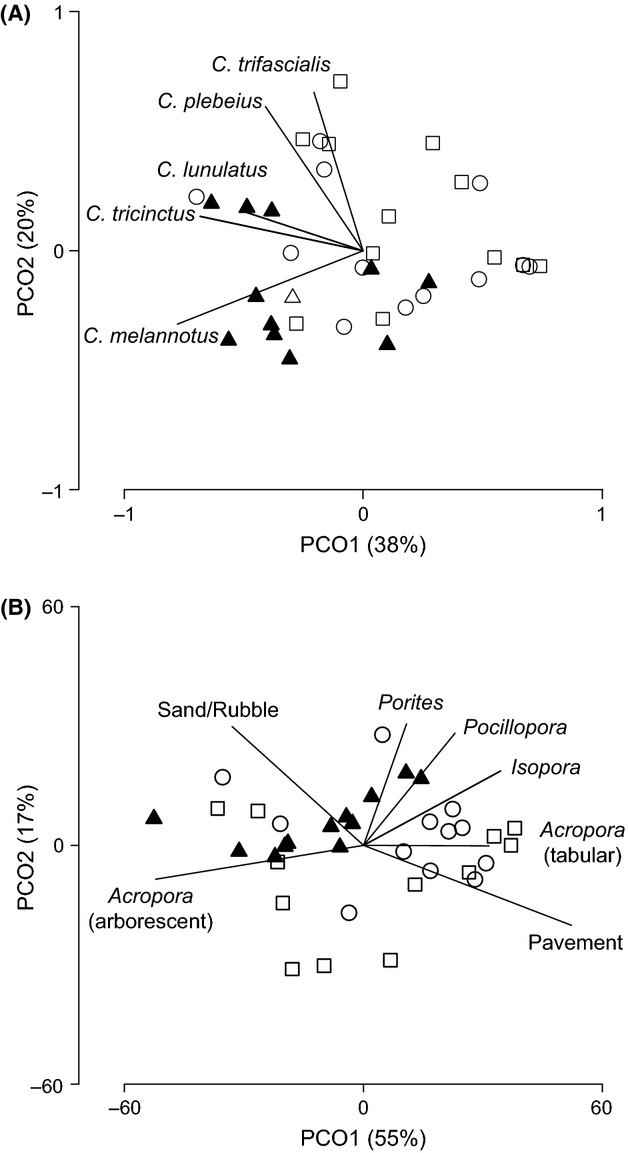
Principal coordinates analysis (PCO) of spatial variation in the abundance and composition of the (A) *Chaetodon* assemblage, and (B) coral reef habitat for 36 transects spread across three sites (North Bay = *filled triangles*, Potholes = *open circles*, Stephen’s Hole = *open squares*) at Lord Howe Island. Vectors are variables (*Chaetodon* species and substratum categories, respectively) most correlated (Pearson’s correlation coefficient, *r* > 0.4) with the PCO axes.

A total of 13 species of *Chaetodon* butterflyfish were recorded across the three lagoonal reef sites at Lord Howe Island, although six of these species (*Chaetodon citrinellus, Chaetodon vagabundus, Chaetodon speculum, Chaetodon ephippium*, *Chaetodon guentheri*, and *Chaetodon pelewensis*) were rare (Fig. 2). Butterflyfish assemblages were significantly different among sites (PERMANOVA: pseudo-*F*_2,33_ = 2.98, *P* = 0.003), largely due to significant differences between North Bay and the other sites (pseudo-*t*_22_ = 1.79, *P* = 0.009 and pseudo-*t*_22_ = 2.30, *P* = 0.001 pairwise comparisons with Potholes and Stephen’s Hole, respectively), with no significant difference between Potholes and Stephen’s Hole (pseudo-*t*_22_ = 0.73, *P* = 0.760). Ordination revealed that spatial variation in *Chaetodon* assemblages was largely due to variation in abundance of five abundant species: *C. tricinctus*, *C. melannotus*, *C. plebeius*, *C. lunulatus,* and *C. trifascialis* (Fig. 3A). Densities of both *C. tricinctus* and *C. melannotus* were 2–3 times higher at North Bay (average = 23.0 and 7.42 fishes per 200 m^2^, respectively) compared to Stephen’s Hole and Potholes.

**Figure 4:**
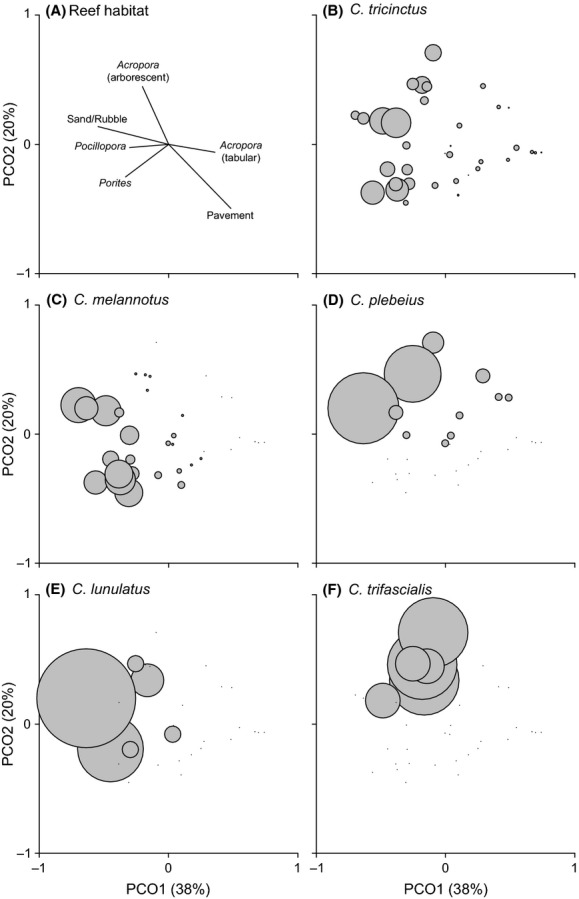
Optimized principal coordinate analysis (PCO) of spatial variation in *Chaetodon* abundance and composition across 36 transects at Lord Howe Island. (A) Reef habitat variables most correlated (Pearson’s correlation coefficient, *r* > 0.4) with the PCO axes. Bubble sizes indicate proportional abundance of (B) *Chaetodon tricinctus*, (C) *Chaetodon melannotus*, (D) *Chaetodon plebeius*, (E) *Chaetodon lunulatus,* and (F) *Chaetodon trifascialis* in areas characterized by tabular *Acropora* and/or pavement (indicated on panel A as transects toward bottom right quadrants of each panel), arborescent *Acropora* (top left quadrants) or *Porites*, *Pocillopora* and sand/rubble (bottom left quadrants).

Similarly, reef substratum composition was significantly different among sites (pseudo-*F*_2,33_ = 3.34, *P* = 0.009), particularly between North Bay and the other two sites (pseudo-*t*_22_ = 2.33, *P* = 0.004 and pseudo-*t*_22_ = 1.87, *P* = 0.034), but not between Potholes and Stephen’s Hole (pseudo-*t*_22_ = 1.27, *P* = 0.176). Spatial variation in reef habitat structure was largely attributable to seven benthic categories: sand/rubble, pavement, *Acropora* (arborescent), *Acropora* (tabular), *Pocillopora*, *Isopora* and *Porites* (Fig. 3B). Cover of scleractinian corals was much higher at North Bay (43.4%) compared to Stephen’s Hole (38.7%) and Potholes (30.3%), mostly because of higher cover of arborescent *Acropora* (32.1%), which was the dominant coral at North Bay (comprised 73.9% of all coral). DISTLM marginal tests indicated scleractinian corals accounted for 46.0% of variation in *Chaetodon* assemblages, followed by abiotic substratum types (sand/rubble and pavement, 20.4%), soft coral and macroalgae (<0.1% each, Table 1A). *Porites*, *Acropora* (arborescent), *Pocillopora,* and/or *Cyphastrea* appear to provide the best explanatory habitat variables in distance-based linear models of spatial variation in the Lord Howe Island *Chaetodon* assemblage (Table 1B). While proportional abundances for each of the above five *Chaetodon* species tended to be highest in areas characterized by some of these types of coral (Fig. 4), considerable variation remains unexplained in these habitat-based DISTLMs (i.e., all *r*^2^ < 0.28, Table 1B).

**Table 1:** Summary of (A) marginal tests and (B) distance-based linear model (DISTLM) selection, based upon Akaike Information Criteria for finite samples (AICc) to select “best” model combinations of habitat variables (i.e., best solutions for models with 1, 2, 3, or 4 variables) to explain spatial variation in *Chaetodon* assemblages at Lord Howe Island. Marginal tests are for higher groupings of substratum variables to explore overall trends in multivariate variation (following Anderson et al. 2008). Abiotic categories (sand/rubble and pavement) were excluded from DISTLM selection due to strong (negative) correlations with biotic categories (following Anderson et al. 2008)

(A) Marginal tests						
Group	SS	Residual df	Regression df	% variation	Pseudo-*F*	*P*
Scleractiniancoral	7.035	23	13	46.0	1.64	0.008
Sand/rubble/pavement	3.123	33	3	20.4	4.24	0.001
Soft coral	0.903	34	2	0.06	2.14	0.063
Macroalgae	0.649	34	2	0.04	1.51	0.145

(B) Best DISTLM solutions			
Habitat variables	AICc	SS (resid.)	*r*^2^
*Porites*	29.16	14.05	0.08
*Porites* + *Acropora* (arborescent)	31.05	12.60	0.18
*Porites* + *Acropora* (arborescent) + *Pocillopora*	30.93	11.78	0.23
*Porites* + *Acropora* (arborescent) + *Pocillopora* + *Cyphastrea*	30.24	11.14	0.27

The three stripe butterflyfish, *C. tricinctus* was by far the most abundant *Chaetodon* species at all locations, accounting for 67.7% of all individuals (Fig. 2). The mean abundance of *C. tricinctus* was 14.08 ± 2.05 (SE) fish per 200 m^2^, compared to 3.72 ± 0.78 SE fish per 200 m^2^ for the next most abundant species, *C. melannotus* (Fig. 2). Most *C. tricinctus* (374 of 640 individuals) occurred in schools of up to 42 individuals, with only 16% of individuals (*n* = 142) recorded in pairs, and 14% of individuals (*n* = 124) observed on their own. Larger aggregations of *C. tricinctus* tended to be found in interreefal habitats (over sand), but in close proximity to colonies of arborescent *Acropora* (Fig. 4B). Abundance of *C. trifascialis* was also highest where there was high arborescent *Acropora* (Fig. 4F), while abundance of *C. lunulatus* was highest where there was high cover of *Pocillopora* (Fig. 4E).

### Feeding behavior

Feeding rates (number of bites taken per 3-min) varied greatly within and among the butterflyfishes considered during this study (*C. lunulatus*, *C. plebeius*, *C. tricinctus*, and *C. trifascialis*). Notably, a large proportion of *C. melannotus* (35 of 67) and *C. tricinctus* (43 of 141) were not seen to take any bites throughout an entire 6-min observation period (i.e., when including the 3-min acclimation period), in contrast to very few (0–2) instances of nonfeeding in the other species. Accordingly, mean bites rates of *C. melannotus* and *C. tricinctus* were markedly lower than *C. lunulatus*, *C. plebeius*, and *C. trifascialis*, with mean bite rates (averaged across all sites) varying by a factor of six among these species (Table 2). Bite rates varied significantly among species, but also varied among sites (Table 3), whereby the feeding rates for all but *C. trifascialis* were higher at Stephen’s Hole than at North Reef or Potholes. For *C. lunulatus*, bite rates recorded at Stephen’s Hole (27.00 bites per 3-min ± 7.22 SE) were twice those recorded at Potholes (12.56 bites per 3-min ± 1.66 SE). For *C. melannotus*, bite rates recorded at Stephen’s Hole (5.36 bites per 3-min ± 2.51 SE) were three times higher than recorded at Potholes (1.72 bites per 3-min ± 0.71 SE) or North Bay (1.78 bites per 3-min ± 0.67 SE). For *C. trifascialis*, bite rates were consistently high across all sites, but were highest at North Bay (19.9 bites per 3-min ± 1.02 SE). Even after accounting for those individuals that did not feed at all, the mean number of bites taken by *C. melannotus* (5.78 bites per 3-min ± 1.69 SE) and *C. tricinctus* (11.98 bites per 3-min ± 0.84 SE) were much lower than for the other three species (Table 2).

**Table 2:** Bite rates, coral use, and feeding selectivity of five *Chaetodon* butterflyfishes at Lord Howe Island, ordered according to increasing selectivity. Significance of prey selection was assessed using forage selection ratios and Bonferroni corrected 95% confidence intervals (“=“indicates prey that were used in proportion to availability, “+” indicates prey used significantly more than expected, “−” indicates prey used less than expected, and “0” indicates prey that were not used at all). Overall significance of feeding selectivity was tested using total forage ratios, comparing relative use of different prey categories to their availability across the three study sites (Manly et al. 2002)

Species	*n*	Bite rate	Hard corals (%)	Arborescent *Acropora*	Tabular *Acropora*	*Isopora*	*Pocillopora*	*Porites*	Soft corals	Total Forage Ratio	Sig.
*Chaetodon melannotus*	67	2.85	6.28	3.14% (−)	1.05% (−)	0.52% (−)	1.05% (−)	0.52% (−)	45.03% (+)	1797.53	<0.001
*Chaetodon lunulatus*	51	16.69	99.76	30.55% (−)	6.46% (+)	3.06% (=)	29.38% (+)	20.92% (+)	0.00% (0)	2849.47	<0.001
*Chaetodon plebeius*	65	15.85	99.90	23.20% (−)	16.21% (+)	26.70% (+)	19.42% (=)	9.81% (+)	0.00% (0)	3499.17	<0.001
*Chaetodon tricinctus*	141	8.33	100	51.57% (=)	22.38% (+)	1.96% (−)	19.23% (=)	0.68% (−)	0.00% (0)	3940.70	<0.001
*Chaetodon trifascialis*	73	17.63	100	55.71% (=)	38.54% (+)	0.39% (−)	3.89% (−)	0.23% (−)	0.00% (0)	4552.50	<0.001

**Table 3:** Two-way factorial ANOVAs testing for differences in (A) bite rates and (B) the range of prey types consumed among species (see Table 2 for details) and among the three distinct study locations (North Bay, Stephen’s Hole and Potholes). Given that both the total number of bites and the number of distinct prey types consumed within a 3-min period is highly constrained, data were square-root transformed prior to analyses

A) Bite rate					
Source	SS	df	MS	*F*	Sig.
Species	518.74	4	129.68	58.95	<0.001
Sites	35.37	2	17.68	8.04	<0.001
Species × sites	23.20	8	2.90	1.32	0.23
Error	840.40	382	2.20		
Total	4534.00	396			

B) Range of prey types					
Source	SS	df	MS	*F*	Sig.
Species	6.58	4	1.64	16.56	<0.001
Sites	0.29	2	0.15	1.49	0.23
Species × sites	0.63	8	0.08	0.79	0.61
Error	30.20	382	0.10		
Total	545.00	396			

**Figure 5:**
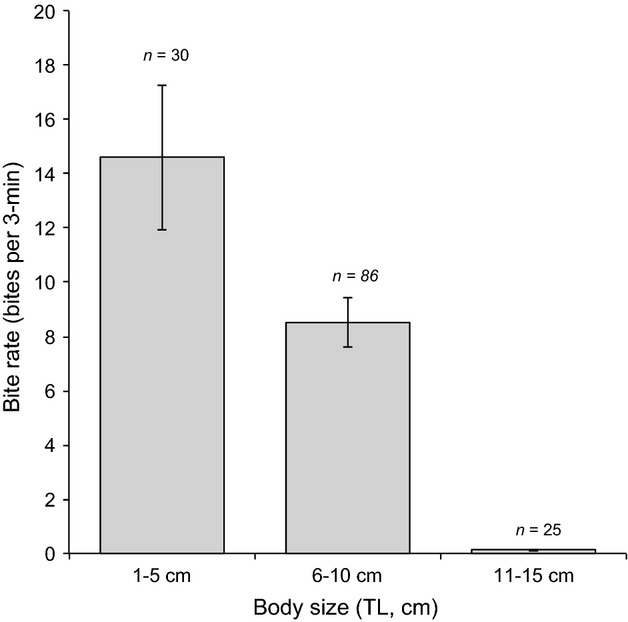
Size-based variation in mean (±SE) bites rates of *Chaetodon tricinctus*. Total length (TL) was visually estimated (to the nearest cm) for all fishes for which 3-min feeding observations were conducted. Data were pooled across site, and the number of fishes within each size class (*n*) is shown.

For *C. tricinctus*, feeding rates differed significantly among fishes in different size classes (ANOVA, *F*_2,138_ = 1434.25, *P* < 0.001), being highest for the smallest fishes (14.60 bites per 3-min ± 2.67 SE) and declining with increasing TL (Fig. 5). All individuals <5 cm TL remained in close proximity to the benthos feeding almost continually on scleractinian corals throughout feeding observations. Among *C. tricinctus* of 6–10 cm TL, 20 individuals (of 86 in total) did not feed; larger individuals that did feed under observation exhibited sustained feeding on scleractinian corals, taking a mean of 11.12 bites per 3-min (±1.05 SE). For individuals >10 cm, only 2 (of 25) individuals were seen to feed on benthic substrata and these fishes took only 1 and 2 bites, respectively, throughout a 3-min observation. For the most part, all individuals >10 cm TL remained in schools in mid-water and rarely approached or searched the substratum during our diurnal observations. While it is possible that they were opportunistically feeding on passing plankton, as they did occasionally open and close their mouths, they tended to move very slowly rather than making any darting movements to actively seek out planktonic prey.

Four (of five) dominant *Chaetodon* butterflyfishes (*C. lunulatus*, *C. plebeius*, *C. tricinctus*, and *C. trifascialis*) at Lord Howe Island were classified as obligate corallivores (following Cole et al. 2008) due to them taking almost 100% of recorded bites from the surface of live corals (Table 2). The exception was *C. melannotus*, which took only 6.28% of bites from the surface of scleractinian corals, with most of their bites taken on soft corals. All of the obligate corallivore species fed predominantly on *Acropora* (Table 2), which was prevalent across all sites. However, all four species of butterflyfishes clearly distinguished between different types of *Acropora*, consuming tabular *Acropora* disproportionately more than expected based on availability across the three sites, while they consumed arborescent *Acropora* in lower or equal proportions to availability (Table 2).

**Figure 6:**
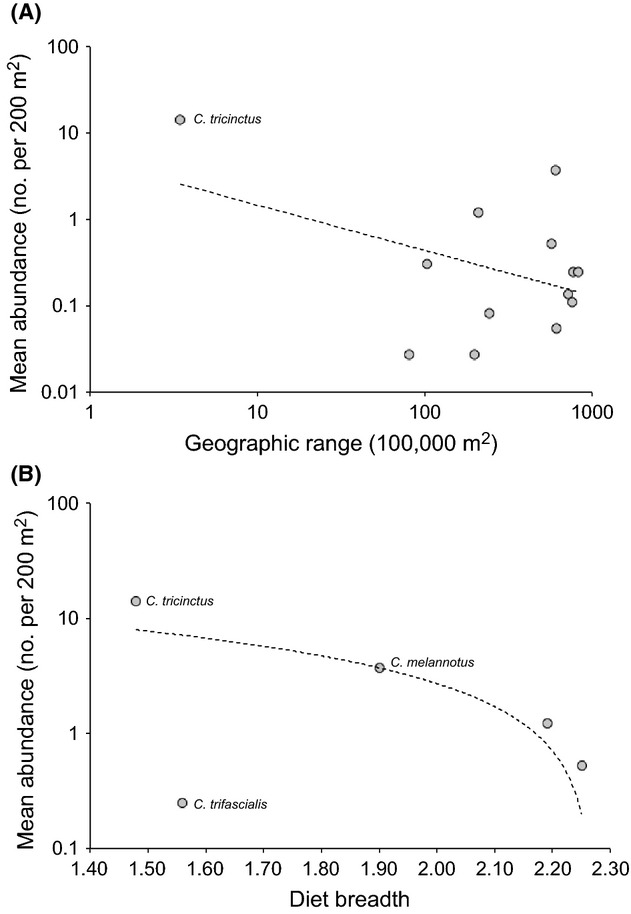
Correlations of (A) geographic range and (B) dietary breadth versus mean abundance (averaged across all sites) for *Chaetodon* butterflyfishes at Lord Howe Island. Abundance and geographic range are shown on a log-scale. Dietary breadth was estimated only for the five most abundant butterflyfishes at Lord Howe Island.

All butterflyfishes exhibited significant levels of dietary selectivity (Table 2), consuming some corals disproportionately to their availability. *Chaetodon melannotus* avoided all scleractinian corals in preference for soft corals (Table 2), but still consumed an average of 1.90 different coral types per 3-min observation (Fig. 6B). *Chaetodon lunulatus* was the least selective of the four obligate corallivores, consuming an average of 2.25 different coral genera per 3-min observation (Fig. 6B). While most bites were taken from arborescent *Acropora*, *C. lunulatus* preferentially consumed tabular *Acropora*, *Porites,* and *Pocillopora* (Table 2). *Chaetodon plebeius* exhibited intermediate levels of dietary selectivity, consuming an average of 2.19 different coral genera per 3-min observation (Fig. 6B) and preferentially consumed preferentially consumed tabular *Acropora*, *Isopora,* and *Porites*. *Chaetodon tricinctus* and *C. trifascilis* were the most specialized coral feeders (Table 2), generally consuming only 1–2 different coral genera during feeding observations. Both species took most bites from arborescent *Acropora*, but preferred tabular *Acropora* to the exclusion of most other coral prey (Table 2), while most strongly avoiding *Isopora* and *Porites*.

### Correlates of species abundance

*Chaetodon tricinctus* was the dominant butterflyfish at all study sites, and while their abundance varied, it tended to be >3 times more abundant than any other butterflyfish species present. Found only at Lord Howe Island, nearby Elizabeth and Middleton Reefs and Norfolk Island, *C. tricinctus* geographic range is <5% of the next smallest range species, *C. uentheri*. The most widespread species recorded at Lord Howe Island, *Chaetodon auriga* and *C. trifascialis*, have widespread geographic ranges that extend across the entire Indo-Pacific and are >200 times larger than that of *C. tricinctus*, but both these widespread species are rare at Lord Howe Island (especially compared to *C. tricinctus*). Mean abundance of coral reef butterflyfishes at Lord Howe Island (averaged across the three sites) was weakly negatively correlated (*r* = −0.40, *n* = 13, *P* = 0.18) with geographic range (Fig. 6A). This relationship appeared to be driven by the high abundance and limited geographic range of *C. tricinctus*. Indeed excluding *C. tricinctus* from the analysis resulted in no relationship between abundance and geographic range (*r* = 0.09, *n* = 12, *P* = 0.78).

Regardless of the metric, *C. trifascialis* and *C. tricinctus* have the most specialized diets at Lord Howe Island. Notwithstanding the apparent lack of feeding among larger individuals, *C. tricinctus* used available coral prey in very similar proportions to *C. trifascialis*, feeding predominantly on arborescent *Acropora*, but selectively targeting tabular *Acropora* (Table 2). The main difference was that *C. trifascialis* avoided eating *Pocillopora* corals, whereas *C*. *tricinctus* consumed *Pocillopora* in approximate accordance with its’ availability. Despite similarities in their selectivity and dietary composition, *C. tricinctus* was >50 times more abundant than *C. trifascialis*, being the most and least abundant (respectively) of the five species for which dietary composition was analyzed. Other coral-feeding butterflyfishes (*C. melannotus*, *C. plebeius,* and *C. lunulatus*) were less selective and less abundant compared to *C. tricinctus*, suggesting that if there was any relationship between mean abundance and diet breadth it would be negative (Fig. 6B). However, the actual relationship based on these five species was nonsignificant (*r* = −0.34, *n* = 5, *P* = 0.58).

## Discussion

The extent to which patterns of local abundance in coral reef fishes can be related to ecological specialization and/or geographical range size is uncertain, given the wide variety of relationships detected among taxonomic groups and locations (e.g., Hawkins et al. 2000; Bean et al. 2002; Hobbs et al. 2010; Berkström et al. 2012). Here, we reveal that marked interspecific variations in the local abundance of coral reef butterflyfishes at Lord Howe Island are weakly correlated to the geographic range size of species, but unrelated to levels of feeding specialization. Much of this range–abundance relationship hinges upon the most abundant species, *C. tricinctus*, which is a regional endemic with >3 times higher abundance than any other butterflyfish species at Lord Howe Island, and is the dominant species across all of our study sites. While high local abundances are often thought to be linked to high levels of preferred resource availability (Brown 1984; Brown et al. 1995; Gregory and Gaston 2000), in *C. tricinctus* we find unusual foraging behavior that is, unlike any other butterfly-fish classed as an obligate corallivore (Cole et al. 2008).

Despite their vulnerability to coral loss (e.g., Pratchett et al. 2006), butterflyfish assemblages are often dominated by obligate coral-feeding species (reviewed by Pratchett 2014). At Lord Howe Island, obligate coral-feeding species (including *C. tricinctus*) accounted for 77.43% of all butterflyfishes (580 of 749), and three of four of the most abundant species were all obligate coral-feeding species. Obligate corallivores also dominate butterflyfish assemblages at many other locations throughout the Indo-Pacific (Emslie et al. 2010; Pratchett et al. 2013a; Cole and Pratchett 2014), but it is less clear to what extent specialist versus generalist corallivores dominate butterflyfish assemblages.

Highly specialized species are expected to be much less abundant than generalist counterparts because they are assumed to be more constrained by a narrower range of possible resources (Brown 1984; Gaston et al. 1997). While such trends have been recorded in some coral reef fishes (Hawkins et al. 2000; Bean et al. 2002), the relative abundance of generalist versus specialists species within a specific location will depend upon the availability of different resources (Munday 2004); consequently, specialist species may be more abundant where their preferred resources are also abundant (Brown 1984; Emslie et al. 2010; Pratchett et al. 2013a). At Lord Howe Island, four species of obligate coral-feeding butterflyfishes (*C. lunulatus*, *C. plebeius*, *C. tricinctus*, and *C. trifascialis*) all consumed tabular *Acropora* disproportionately to its availability, as shown elsewhere (Berumen and Pratchett 2006; Cole et al. 2012; Pratchett et al. 2013a). Given that proportional consumption of tabular *Acropora* was highest for the two most specialized species, *C. tricinctus* and *C. trifascialis* (Table 2), it may be that a predominance of *Acropora* corals at Lord Howe Island (which accounted for up to 94% of coral recorded on individual transects) confounds the expected negative relationship between dietary specialization and abundance. While it is clear that specialist butterflyfishes are numerically dominant in some locations (e.g., Pratchett et al. 2013a), this is not necessarily the case at Lord Howe Island. The dominant species, *C. tricinctus*, does feed on a relatively restricted range of different corals, but it is not altogether clear how this species derives sufficient energy, especially as adults.

While it has long been assumed that *C. tricinctus* consumes mainly scleractinian corals (Kuiter 1996), which is consistent with its’ abundance in coral-rich habitats (Lieske and Myers 2001; Hobbs et al. 2009; Hoey et al. 2014), this is the first detailed study of their foraging behavior. Based on phylogenetically conserved patterns of feeding (e.g., Bellwood et al. 2010) one would assume *C. tricinctus* is an obligate corallivore. Bellwood et al. (2010) showed that *C. tricinctus* is within a clade containing all obligate hard-coral-feeding butterflyfishes. Clearly, when *C. tricinctus* feeds on corals (e.g., as juveniles) it is very selective, and preferentially targets *Acropora* and *Pocillopora*. Bite rates of small (<5 cm TL) *C. tricinctus* (14.60 bites per 3-min ± 2.67 SE) are also consistent with bite rates recorded for other obligate coral-feeding butterflyfishes (Gregson et al. 2008). However, the adult foraging behavior is very different to other obligate coral-feeding butterflyfishes. Obligate coral-feeding butterflyfishes typically exhibit sustained high levels of diurnal feeding upon hard corals (Gregson et al. 2008), which is attributed to physical constraints on the amount of coral tissue that can be effectively removed with each bite (Tricas 1989). It is possible that cooler water temperatures at this high-latitude coral reef may be reducing metabolic rates and altering the energetic budgets of these tropical fishes (Beamish 1981; Harmelin-Vivien 2002; Pörtner 2002), which may manifest as different types of foraging behaviors among these butterflyfish species (Clarke 2003). Size-based declines in feeding rates have been recorded among other functional groups of fishes (e.g., van Rooij et al. 1996; Bonaldo et al. 2006), and may reflect declines in energetic requirements among large and mature individuals, whereas juveniles invest substantially into growth and development (Harmelin-Vivien 2002). It is also possible that adult *C. tricinctus* feed mainly at night, as has been suggested for some other coral-feeding butterflyfishes (Zekeria et al. 2002). Alternatively, *C. tricinctus* may fundamentally alter its foraging behavior with ontogeny, as shown for some coral-feeding wrasses (Cole 2010).

The schooling behavior of *C. tricinctus* is also very unique, especially among corallivorous butterflyfishes. Aside from Lord Howe Island, we know that *C. tricinctus* is also very abundant and often forms large schools at Elizabeth and Middleton Reefs (Hobbs et al. 2009; Hoey et al. 2014), but is generally rare and occurs singly or in pairs at Norfolk Island (van der Meer et al. 2013). In reviewing the social organization of butterflyfishes, Hourigan (1989) reported that schooling is restricted to planktivorous butterflyfishes, whereas obligate corallivores tend to form pairs that aggressively maintain distinct feeding territories (Hourigan 1989; Roberts and Ormond 1992). Schooling behavior among coral reef fishes is generally considered to be a strategy to decrease search times for patchily distributed resources, provide increased protection from predators, and/or save on the energetic costs of locomotion (Ward et al. 2002; Liao 2007; Pereira and Ferreira 2013). Without further evidence (e.g., observations of nocturnal behavior) it is difficult to conclude whether this behavior plays a role in driving the extreme abundance of *C. tricinctus* at Lord Howe Island (especially, compared to other butterflyfishes).

Aside from resource use and availability, interspecific differences in abundance of coral reef fishes may be explained by contrasting population dynamics and key demographic rates. In particular, the relative abundance of different fishes is fundamentally dependent upon species-specific rates of recruitment (e.g., Schroeder 1987; Doherty and Williams 1988; Doherty 1991; Caselle and Warner 1996) and this is likely to be even more important at relatively isolated locations, such as Lord Howe Island. Small and isolated coral reefs, like islands, often contain a high proportion of endemic species (Jones et al. 2002; Allen 2008). Moreover, endemic marine fishes are often more (not less) abundant than their widespread counterparts (e.g., Hourigan and Reese 1987; Randall 1998; Jones et al. 2002; DeMartini 2004; DeMartini and Friedlander 2004; Hobbs et al. 2010, 2011). One obvious explanation for this pattern is that restricted range species have reproductive strategies that minimize dispersal and advection of larvae away for their natal reefs, thereby limiting the capacity for range expansion, but also ensuring effective self-recruitment (e.g., DeMartini 2004; DeMartini and Friedlander 2004; Eble et al. 2009; Hobbs et al. 2011). Consistent with this hypothesis, we recorded few (if any) very small (<5 cm TL) individuals, assumed to represent new recruits, for any species, except *C. tricinctus*. Moreover, van der Meer et al. (2013) showed that there are very high rates of self-recruitment at each of the reefs (Lord Howe Island, Elizabeth and Middleton Reefs) where *C. tricinctus* is the predominant butterflyfish species. However, interspecific comparisons of recruitment rates will require systematic surveys over multiple recruitment seasons, as well detailed demographic studies to account for possible interspecific differences in growth rates.

There is increasing evidence that terrestrial macroecological relationships between abundance and range size do not necessarily apply to coral reef fishes (e.g., Hobbs et al. 2010, 2011, 2012; Berkström et al. 2012). Contrary to expectations, the most abundant species of butterflyfish at Lord Howe Island, *C. tricinctus*, is a restricted range endemic and also appears to be among the most specialized of butterflyfishes recorded at this location. Endemic species may predominate at isolated locations because they are uniquely adapted to the local conditions (Blackburn et al. 1997; Thiollay 1997; Reif et al. 2006). Similarly, highly specialized species may be particularly abundant at locations with very high availability of their preferred habitat and/or food resources. *Chaetodon tricinctus*, however, remains an enigmatic species that contradicts much of the established understanding of coral-feeding butterflyfishes. Future research needs to consider whether the energetic demands (metabolic rates) of *C. tricinctus* are fundamentally different from that of other coral-feeding butterflyfishes, or how adult fishes derive necessary energy despite infrequent bouts of benthic feeding. This research is necessary to clearly establish the vulnerability of *C. tricinctus* to increasing degradation of coral reef environments. Specialist coral-feeding butterflyfishes are extremely vulnerable to sustained and ongoing coral loss (Pratchett et al. 2008) that is, occurring on reefs throughout the world (Hughes et al. 2003), but flexible foraging (Noble et al. 2014) and highly resilient population dynamics may help to buffer against species extinctions (Lawton et al. 2011).
